# Oyster (*Crassostrea gigas*) Hydrolysates Produced on a Plant Scale Have Antitumor Activity and Immunostimulating Effects in BALB/c Mice

**DOI:** 10.3390/md8020255

**Published:** 2010-02-02

**Authors:** Yu-Kai Wang, Hai-Lun He, Guo-Fan Wang, Hao Wu, Bai-Cheng Zhou, Xiu-Lan Chen, Yu-Zhong Zhang

**Affiliations:** 1 State Key Laboratory of Microbial Technology, Marine Biotechnology Research Center, Shandong University, Jinan, 250100, China; E-Mails: dawson82@sina.com (Y.-K.W.); helenhe@sdu.edu.cn (H.-L.H.); wuhaojr@sina.com (H.W.); bczhou@ms.qdio.ac.cn (B.-C.Z.); zhangyz@sdu.edu.cn (Y.-Z.Z.); 2 Shandong Cancer Hospital, Jinan, 250117, China; E-Mail: gf-wang@21cn.com (G.-F.W.)

**Keywords:** hydrolysates, oyster, immunostimulating peptide, antitumor, BALB/c mice

## Abstract

Oyster extracts have been reported to have many bioactive peptides. But the function of oyster peptides produced by proteolysis is still unknown. In this study, the oligopeptide-enriched hydrolysates from oyster (*Crassostrea gigas*) were produced using the protease from *Bacillus* sp. SM98011 at laboratory level, and scaled up to pilot (100 L) and plant (1,000 L) levels with the same conditions. And the antitumor activity and immunostimulating effects of the oyster hydrolysates in BALB/c mice were investigated. The growth of transplantable sarcoma-S180 was obviously inhibited in a dose-dependent manner in BALB/c mice given the oyster hydrolysates. Mice receiving 0.25, 0.5 and 1 mg/g of body weight by oral gavage had 6.8%, 30.6% and 48% less tumor growth, respectively. Concurrently, the weight coefficients of the thymus and the spleen, the activity of natural killer (NK) cells, the spleen proliferation of lymphocytes and the phagocytic rate of macrophages in S180-bearing mice significantly increased after administration of the oyster hydrolysates. These results demonstrated that oyster hydrolysates produced strong immunostimulating effects in mice, which might result in its antitumor activity. The antitumor and immunostimulating effects of oyster hydrolysates prepared in this study reveal its potential for tumor therapy and as a dietary supplement with immunostimulatory activity.

## 1. Introduction

Enzymatic hydrolysis is an attractive method for modifying the physical properties of food proteins to improve their nutritional properties [1]. The application of enzyme technology to recover modified food proteins may produce a broad spectrum of food ingredients or industrial products [2–4]. Recent studies have demonstrated the capacity of enzymes to produce novel food products, modify foodstuffs and improve waste management [1,5–7].

In recent years, opportunistic or complicated infections often lead to stress-associated immunosuppression, which are difficult to treat with antibiotics alone and continue to be a challenge in the clinical field [8]. Moreover, most immunomodulatory pharmaceuticals are not suitable for chronic or preventive use. Therefore, there is an increasing interest in identifying new immunomodulators to enhance nonspecific host defense mechanisms [8]. Immune peptides are a group of extremely diverse small proteins that are suitable as biocontrol agents because they: (i) have a broad spectrum of target microorganisms, including bacteria, fungi and viruses; (ii) have not, to date, induced pathogen resistance; (iii) are generally non-toxic, and can meet food safety requirements. Many immune peptides have been reported in hydrolysates from food proteins digested with proteases [9], but most of them are from terrestrial proteins rather than from marine sources. The composition and primary amino acid sequences of marine proteins are different from those of terrestrial proteins. Therefore, marine proteins may be an important resource for novel immune peptides following enzymatic hydrolysis. Indeed, peptides isolated from fish hydrolysates show strong immunostimulating effects [10,11]. The immunostimulant activity of enzymatic protein hydrolysates from microalgae *Chlorella* has also been demonstrated in mice [12]. Pulz and Gross reported that the world production of *Chlorella* amounts to 2,000 ton/year and the product is applied in the protein enrichment of functional foods and nutritional supplements [13].

Oyster proteins are a potential resource for active bio-peptides. It has been reported that oyster extracts have many bioactive peptides, such as ACE (angiotensin-converting enzyme) inhibitory peptides [14], anti-fungal peptides [15] and anti-cancer peptides [16]. Thus, oyster proteins are likely to contain a large amount of various bioactive sequences. These bioactive sequences can be released by enzyme hydrolysis to serve as bioactive peptides, and therefore, enzyme hydrolysis may improve the nutritional properties of oyster proteins.

In this study, oyster (*Crassostrea gigas*) hydrolysates were prepared with the protease from *Bacillus* sp. SM98011 and production was scaled up to pilot and plant scales. The antitumor activity and immunostimulating effects of plant-scale oyster hydrolysates on S180-bearing BALB/c mice were investigated. The results showed that the oyster hydrolysates had obvious antitumor activity and enhanced immune function in BALB/c mice, implying that the oyster hydrolysates may have potential in tumor therapy and as a dietary supplement with immunostimulatory activity.

## 2. Experimental Section

### 2.1. Materials

Pacific oysters (*C. gigas*) were bought at a local fish market. Protease was prepared from *Bacillus* sp. SM98011 and an activity assay was performed using the methods previously described [17]. Concanavalin A (Con A), Wright-Giemsa stain and 3-(4,5-dimethylththiazoyl-2-yl)-2,5- diphenyltetrazolium bromide (MTT) were purchased from Sigma (USA). RPMI-1640 and fetal bovine serum (FBS) were purchased from Gibco (USA). Mouse lymphadenoma YAC-1 cell lines (sensitive to NK cells), and S180 mouse sarcoma cell lines were purchased from Shandong Academy of Medical Sciences, China.

### 2.2. Production of oyster hydrolysates on a laboratory scale and its scale-up to pilot and plant levels

Hydrolysis of the oysters was performed on a laboratory scale (500 mL Erlenmeyer flask), pilot scale (100 L thermostatically stirred-batch reactor) and plant scale (1,000 L thermostatically stirred-batch reactor). For the laboratory scale, a reaction mixture consisting of 50 g of minced oysters and 10 mL crude protease solution (400,000 U/kg, enzyme/fresh weight of protein substrate) in a 500 mL Erlenmeyer flask was adjusted to pH 7.5 with NaOH or HCl. The reactor vessel was placed in a thermostatically controlled water-bath with constant agitation (200 rpm) at 50 °C. After 5 h, the reaction was stopped with a 15-min incubation at 90 °C [18]. The resulting slurry was centrifuged at 9,000 ×*g* for 20 min at 4 °C. The supernatant was lyophilized and stored at 4 °C until further analysis. For the pilot and plant scales, minced oysters were mixed with water at a ratio of 1:1 (w/w) by continuous stirring and the enzyme was added with 400,000 U/kg. All reactions were performed at pH 7.5 and 50 °C for 5 h with constant agitation (200 rpm). The reactions were terminated by heating the solution to 90 °C for 15 min, assuring the complete inactivation of the enzyme. The resultant slurry was centrifuged at 9,000 ×*g* for 20 min and the supernatant was oyster hydrolysates. The hydrolysates from pilot and plant scales were condensed with a ball vacuum concentration tanker (RD500, GuanYi Mechanical Equipment Co., Ltd, China) and were dried with a spray dryer (BoDa Co., China) at a 10 kg/h flow rate with a 170 °C inlet temperature and 90 °C outlet temperature [19].

### 2.3. Characterization of oyster hydrolysates

Assays of the peptides and amino acids in the hydrolysates were performed using the methods previously described [20]. The peptides larger than 3 kDa were removed by ultrafiltration with an ultrafiltration membrane (molecular weight cut-off 3 kDa). Fraction analysis of the peptides in the hydrolysates was performed using reversed phase high-performance liquid chromatography (RP-HPLC, Waters alliance 2695, USA) coupled with a Dual Wavelength UV Detector 2487 on a Symmetry (Waters, Milford, MA, USA) C18 column (250 mm × 4.6 mm). Separation was performed with a linear gradient of acetonitrile from 6.7% to 40% containing 0.1% trifluoroacetic acid for 30 min followed by a linear gradient of methanol from 40% to 6.7% for 15 min at a flow rate of 1 mL/min. Peptides were monitored at 214 nm.

### 2.4. Animals

Seventy female BALB/c mice (20 ± 2 g, 6–8 weeks old) were purchased from the Animal Center, Medical Institute of Shandong University, China. The mice were housed under normal laboratory conditions (21 ± 2 °C) with free access to standard rodent chow and water. To transfer the tumor cells to the mice, 0.2 mL of S180 cell suspension (5 × 10^6^ cells/mL) was subcutaneously inoculated into a mouse armpit. The mice were divided into 7 groups of 10 animals each: normal control group, S180-bearing control group, 0.25, 0.5 and 1 mg/g oyster hydrolysates (ultrafiltered) treated groups, 1 mg/g crude oyster hydrolysates (not ultrafiltered) treated group and 0.02 mg/g cyclophosphamide (CTX) treated group. The oyster hydrolysates were administrated by oral gavage once a day for 14 d in the treated groups. Normal control and S180-bearing control groups received the same volume of physiological brine. The CTX treated group was treated with the standard anti-cancer drug cytoxan by intraperitoneal injection. On the 15^th^ day, all the animals were sacrificed and the mice, thymuses, spleens and tumors were weighed. The present study was performed in accordance with the Guidelines in the Care and Use of Animals and was approved by the Center for New Drugs Evaluation of Shandong University’s review committee and Shandong University’s Animal Ethics Committee.

### 2.5. Analysis of the spleen and thymus indices of the mice

The spleens and thymuses were eviscerated out of the mice and weighed to obtain the indices of the spleen and thymus. The spleen index (mg/g) = spleen weight/body weight, and the thymus index (mg/g) = thymus weight/body weight. The antitumor activity of oyster hydrolysates was expressed as an inhibition ratio calculated as [(*A*−*B*)/*A*] × 100%, where *A* and *B* were the average tumor weights of the control and treated groups, respectively.

### 2.6. Assay of lymphocyte proliferation

The MTT test was used to measure lymphocyte proliferation following the method described by Denizot and Lang [21] with modifications. Spleens were aseptically removed from sacrificed mice with scissors and forceps and put in cold phosphate-buffered saline (PBS), gently homogenized and filtered with a fine nylon mesh to obtain single cell suspensions. Then the cells were suspended at a final density of 5 × 10^6^ cells/mL in RPMI-1640 medium supplemented with 10% newborn bovine serum. Subsequently spleen cells (200 μL/well) were seeded into a 96-well plate. Con A (7 μg/mL) as a T cell mitogen was added to the cell suspension and the plate was incubated at 37 °C with 5% CO_2_. After incubation for 72 h, 10 μL MTT (5 mg/mL) was added to each well and the plate was further incubated for 4 h. The plate was centrifuged at 200 ×*g* for 10 min and the supernatants were removed. Dimethyl sulfoxide (DMSO, 100 μL) was added to each well and the plate was shaken until the crystals dissolved. The absorbance of each well was detected at 570 nm on a microplate reader (Multiscan MK3, Thermo Labsystems). The proliferation of lymphocytes = *A*−*B*, where *A* was the OD_570_ of the wells with Con A, and *B* was the OD_570_ of the wells without Con A.

### 2.7. Assay of NK cell activity

NK cell activity was measured following the method of Yuan *et al*. [22]. YAC-1 cells were used as target cells (T) and seeded in 96-well U-bottom culture plates at 1 × 10^4^ cells/well in RPMI-1640 medium. Spleen cells prepared as described above were used as the effector cells (E) and were added at 5 × 10^5^ cells/well to give an E/T ratio of 50:1. The plates were incubated at 37 °C for 20 h in a 5% CO_2_ atmosphere. Then 10 μL MTT (5 mg/mL) was added to each well. The plate was incubated for another 4 h and subjected to an MTT cellular assay. Three kinds of control measurements were performed: a target cell control, a blank control and an effector cell control. NK cell activity was calculated using the following equation:

$$\text{NK cell activity }(\%)=\frac{{\text{OD}}_{\text{T}}-({\text{OD}}_{\text{S}}-{\text{OD}}_{\text{E}})}{{\text{OD}}_{\text{T}}}\times 100\%$$

where OD_T_ is the optical density value of the target cell control. OD_S_ is the optical density value of the test samples, and OD_E_ is the optical density value of the effector cell control.

### 2.8. Assay of macrophage phagocytosis

Macrophage phagocytosis was assessed following the method described by Wang *et al*. [23] with modifications. First, peritoneal exudate macrophage were harvested from BALB/c mice 3 d after intraperitoneal injection of 1 mL of 2% aqueous starch suspension, and were collected into sterile 2 mL tubes. Chicken erythrocytes (0.5 mL, 1% in Hanks’ solution) and 0.5 mL of mouse macrophage were mixed. A sample of the resulting suspension (0.5 mL) was spread on glass slides and incubated for 20 min in a 37 °C, 5% CO_2_ incubator. After incubation, the glass slides were washed with saline to remove unattached cells. Then the slides were treated with methyl alcohol for 5 min and dyed with 4% (v/v) Giemsa-PBS buffer solution. The number of macrophage that ingested chicken erythrocytes was counted under a microscope. The data are presented as the phagocytic rate (%) (macrophage that ingested chicken erythrocytes/total macrophage) and the phagocytic index (ingested chicken erythrocytes/total macrophage).

### 2.9. Statistical analysis

Data are presented as the arithmetic mean ± standard deviation (SD) of triplicate samples. Statistical analysis was performed using SPSS 10.0. Statistical analysis (Student’s *t*-test) was based on comparisons between the S180 control group and the treated groups. P values less than 0.05 were considered statistically significant.

## 3. Results

### 3.1. Production and characterization of the oyster hydrolysates generated on laboratory, pilot and plant scales

Oyster hydrolysates were first produced with crude protease SM98011 on a laboratory scale. The peptide content in the oyster hydrolysates prepared on a laboratory scale was 62.4% and total amino acid content was 67.6%. Then oyster hydrolysate production was scaled up to pilot (100 L) and plant (1,000 L) levels. In pilot- and plant-scale product, the peptide content was 58.6% and 57.9%, and the total amino acid content was 67.8% and 69.8%, respectively. These results were similar to the laboratory scale (Table 1). Therefore, after production was increased to the pilot- and plant-scale levels, the properties and the compositions of the oyster hydrolysates remained fairly consistent with those on a laboratory scale.

**Figure 1:**
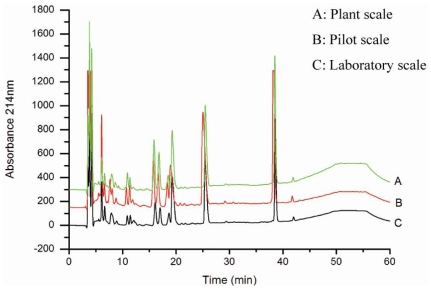
Peptide spectra of oyster hydrolysates produced on laboratory, pilot and plant scales. Hydrolysates were analysed by RP-HPLC on a C18 column under the conditions described in materials and methods.

**Figure 2:**
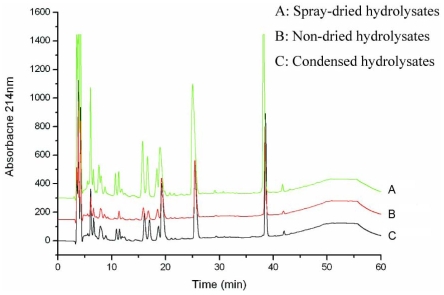
Effects of spray drying on the peptide composition of the oyster hydrolysates. Hydrolysates were analyzed by RP-HPLC on a C18 column under the conditions described in materials and methods.

The peptide spectra of the products from laboratory, pilot and plant volumes were analyzed with RP-HPLC chromatography. The result showed that the peptide spectra of the hydrolysates at pilot and plant scales were identical to those from the laboratory scale (Figure 1). Therefore, the optimized conditions for oyster hydrolysate production on a laboratory scale were well suited for pilot- and plant-scale production. Hydrolysates produced on a plant scale were condensed with a vacuum evaporator and then were dried with a spray dryer. The dried hydrolysate powder dissolved easily in water and its solution was clear with a thin orange-green color. HPLC analysis revealed no significant differences between the non-dried hydrolysates and the dried powder (Figure 2), indicating that exposing the hydrolysates to high temperature did not destroy the composition of the peptides. Therefore, the oligopeptide-enriched hydrolysates from oysters were heat stable.

### 3.2. Antitumor activity of oyster hydrolysates on S180-bearing mice and their effects on thymus and spleen indices

Treatment with 0.5 and 1 mg/g plant-scale hydrolysates significantly inhibited the growth of transplanted sarcoma S180 cells with inhibitory rates of 30.6% and 48%, respectively. Dose-dependent inhibition of tumor growth is shown in Table 2. Meanwhile, all the groups treated with oyster hydrolysates had a significant increase in the thymus and spleen indices compared with S180 control group, indicating that oyster hydrolysates may function as an immunostimulator in S180-bearing mice. In contrast, the chemotherapy drug, CTX had a noticeable inhibitory effect on mouse transplanted sarcoma S180, but also significantly decreased the spleen and the thymus indices (Table 2). These results suggest that oyster hydrolysates probably inhibited the growth of tumor cells by improving the immune function in S180-bearing mice.

### 3.3. Effects of oyster hydrolysates on cellular immunity in S180-bearing mice

Tumor cell elimination is known to be mediated in part by the cytotoxic activity of NK cells [22]. Therefore, the cytotoxic activity of splenocytes against NK-sensitive tumor cells (YAC-1) was measured. As shown in Table 3, spleen lymphocyte proliferation and NK cell activity were significantly decreased in the group treated with CTX in comparison with S180 control group. In contrast, in all 3 groups treated with different amounts of oyster hydrolysates, NK cell activity was significantly increased, and lymphocyte proliferation was also obviously increased in the mid- and high-dose groups. Therefore, oyster hydrolysates enhanced cell-mediated immunity in S180-bearing mice.

### 3.4. Effects of oyster hydrolysates on macrophage phagocytosis in S180-bearing mice

Phagocytosis by macrophage is one of the most important nonspecific immune responses in the human body. The effects of oyster hydrolysates on macrophage phagocytosis were investigated by measuring the phagocytic rate and phagocytic index. Compared with the S180 control group, CTX treatment markedly decreased the phagocytic activity and phagocytic index, while oyster hydrolysates significantly increased the phagocytic rate (p < 0.05) in a dose-dependent manner in S180-bearing mice (Table 4). However, none of the 3 doses of oyster hydrolysates had a significant effect on the phagocytic index. This result indicated that the increased phagocytic activity caused by oyster hydrolysates in S180-bearing mice resulted from an increase in the number of total phagocytes, rather than the activation of phagocytes.

## 4. Discussion

Oyster extracts have been consumed since the 1970s as a dietary supplement, particularly in Japan. They chiefly contain transient elements including iron, copper and zinc, as well as amino acids, including high concentrations of glutamic acid and taurine [24]. Over the years most of the studies on oysters have focused on the biological activities of oyster extracts. It has been reported that oyster extracts can stimulate glutathione expression [25], significantly enhance IL-2 dependent T-cell proliferation [24], and exhibit antioxidant characteristics [26,27]. A peptide that inhibits HIV-1 protease was also isolated from *C. gigas* hydrolysates [28]. However, reports on the immunomodulating activity of oyster protein hydrolysates produced by enzyme technology are rare. As dietary proteins, oyster proteins are likely to contain a large amount of bioactive sequences, which may serve as a potential resource for immunomodulators. In this study, we investigated the immune potential of oyster hydrolysates for the first time. Bioactive hydrolysates of oysters produced with the protease from *Bacillus* sp. SM98011 were produced on a laboratory scale and successfully scaled up to pilot and plant levels. Oral administration of the oyster hydrolysates produced on a plant scale markedly inhibited S180 tumor cell growth, and enhanced immune function in S180-bearing mice.

The relative weights of the spleen and thymus are important indices of nonspecific immunity. It was reported that oral administration of immunopotentiators increased the weights of spleens and thymuses in S180-bearing mice [29]. Our results showed that the relative weights of spleens and thymus increased significantly in a dose-dependent manner in the mice treated with oyster hydrolysates, suggesting that oyster hydrolysates probably function as an immunostimulator.

Immune responses, especially cellular immunity, play an important role in the elimination of tumor cells, and thus inhibit tumor growth. Cell-mediated immune defence is mediated specifically by T cells including NK cells [30]. In S180-bearing mice treated with oyster hydrolysates, there was a significant increase in NK cell activity while tumor growth was inhibited, suggesting that the increased NK cell activity caused by oyster hydrolysates played an important role in eliminating tumor cell growth. Lymphocyte proliferation is also an important indicator of immunoactivation. Stimulating lymphocytes with ConA *in vitro* may be used to evaluate T lymphocyte activity [31]. Oral administration of oyster hydrolysates caused a significant increase in lymphocyte proliferation in the mid and high-dose groups of S180-bearing mice, indicating that oyster hydrolysates enhanced the mitogenic activity of splenic lymphocytes by functioning as a T-cell adjuvant. Bovine lactoferrin hydrolysates have also been reported to induce an antigen-specific proliferative response in T lymphocytes and NK cells [32]. This response may be due to cytokines released from cells in the mucosa of the small intestine [33].

Macrophage play an important role in making innate and adaptive immunities function cooperatively and interdependently [34,35]. Phagocytosis is one of the most important nonspecific immune responses of the body. Therefore, stimulating macrophage is a major target for therapeutic applications [36]. Our results showed that oyster hydrolysates significantly enhanced the phagocytic rates of macrophage, but had no obvious effect on the phagocytic index. Duarte *et al*. reported that fish protein hydrolysates increased the percentage of phagocytosis by peritoneal macrophages in mice [37].

As a well known antitumor drug, CTX has a high tumor inhibitory rate. However, our results and other reports have shown that CTX also has a strong immunosuppressive effect [31]. In contrast, oyster hydrolysates inhibited tumor growth by improving the immune function in S108-bearing mice, which suggests its potential in tumor therapy. In addition, oyster hydrolysates have potential as a dietary supplement that can act as an immunostimulant or as an adjuvant to improve immune function *in vivo*. Since the oyster hydrolysates used in this study were not further purified beyond ultrafiltration, high doses were needed to cause immunomodulating activities in the mice. The potent immunostimulating components in the oyster hydrolysates are being purified to evaluate their potential value in the treatment of human cancers.

## 5. Conclusions

The function of oyster peptides produced by proteolysis is still unknown. In this study, the oligopeptide-enriched hydrolysates from oyster were produced using the protease from *B.* sp. SM98011 at laboratory level, and scaled up to pilot (100 L) and plant (1,000 L) levels. The antitumor activity and immunostimulating effects of the oyster hydrolysates produced at plant scale in BALB/c mice were investigated. The growth of transplantable sarcoma-S180 was obviously inhibited in a dose-dependent manner in BALB/c mice given the oyster hydrolysates. Concurrently, the weight coefficients of the thymus and the spleen, the activity of natural killer (NK) cells, the spleen proliferation of lymphocytes and the phagocytic rate of macrophages in S180-bearing mice significantly increased after administration of the oyster hydrolysates. These results indicated that oyster hydrolysates produced strong immunostimulating effects in mice, resulting in its antitumor activity. The antitumor and immunostimulating effects of oyster hydrolysates prepared in this study reveal its potential for tumor therapy and as a dietary supplement with immunostimulatory activity.

## Figures and Tables

**Table 1:** Amino acid compositions of oyster hydrolysates produced at different scales.

Amino acid	Lyophilized hydrolysates at laboratory scale (g/100 g)	Spray dried hydrolysates at pilot scale (g/100 g)	Spray dried hydrolysates at plant scale (g/100 g)
Asp	6.26	6.39	6.63
Ser	2.69	2.75	2.98
Glu	9.15	9.24	9.27
Gly	3.85	3.66	3.51
His	1.71	1.65	1.77
Arg	7.98	8.40	8.12
Thr	2.82	2.72	2.76
Ala	3.70	3.67	3.86
Pro	2.74	2.76	2.97
Cys	0.23	0.22	0.21
Tyr	0.65	0.65	0.76
Val	1.52	1.55	1.64
Met	3.82	3.82	3.94
Lys	4.24	4.21	4.42
Ile	3.91	3.96	4.17
Leu	6.17	6.30	6.53
Phe	6.15	5.85	6.28
Total	67.59	67.80	69.82

**Table 2:** Effects of oyster hydrolysates on the transplanted tumors and thymus and spleen indices in S180-bearing mice.

Groups	Tumor weight (g)	Inhibition rate (%)	Thymus index (mg/g)	Spleen index (mg/g)
Normal control	-	-	0.6480 ± 0.1143*	6.0961 ± 0.3930***
CTX	0.449 ± 0.118***	82.5	0.5953 ± 0.0565**	5.6334 ± 1.0100**
S180 control	2.567 ± 0.077	-	0.8154 ± 0.0740	8.8186 ± 0.3967
a 0.25 mg/g	2.393 ± 0.111	6.8	0.6969 ± 0.1410	9.4700 ± 1.1800
a 0.5 mg/g	1.781 ± 0.226**	30.6	0.8665 ± 0.0100	9.4267 ± 0.7975
a 1 mg/g	1.335 ± 0.066***	48.0	1.0014 ± 0.0898*	10.7345 ± 0.7030**
Crude b (1 mg/g)	1.922 ± 0.216**	25.1	0.9397 ± 0.1937**	10.2135 ± 0.6570*

Values are presented as mean ± SD.

* Significantly different from S180 control group at P < 0.05.

** Significantly different from S180 control group at P < 0.01.

*** Significantly different from S180 control group at P < 0.001.

a Ultrafiltered hydrolysates;

b Not ultrafiltered hydrolysates.

**Table 3:** Effects of oyster hydrolysates on spleen lymphocyte proliferation and NK cells activity in S180-bearing mice.

Groups	NK activity (%)	Lymphocyte proliferation A_570_
Normal control	42.22 ± 12.60	0.6800 ± 0.0735*
CTX	20.70 ± 6.76*	0.1589 ± 0.0868**
S180 control	29.37 ± 8.83	0.5070 ± 0.0558
a 0.25 mg/g	43.64 ± 9.70*	0.7084 ± 0.1807
a 0.5 mg/g	51.65 ± 9.69**	0.8219 ± 0.0256***
a 1 mg/g	58.35 ± 12.50***	0.8640 ± 0.0456***
Crude b (1 mg/g)	44.30 ± 12.10*	0.7536 ± 0.1196*

Values are presented as mean ± SD.

* P < 0.05.

** P < 0.01.

*** P < 0.001.

a Ultrafiltered hydrolysates;

b Not ultrafiltered hydrolysates.

**Table 4:** Effects of oyster hydrolysates on macrophage phagocytosis in S180-bearing mice.

Groups	Phagocytic rate (%)	Phagocytic index
Normal control	50.0 ± 3.6	1.077 ± 0.045
CTX	37.3 ± 1.5*	0.650 ± 0.036*
S180 control	46.0 ± 1.0	1.130 ± 0.061
a 0.25 mg/g	45.7 ± 1.5	1.073 ± 0.059
a 0.5 mg/g	54.3 ± 2.1*	0.970 ± 0.070
a 1 mg/g	61.0 ± 3.6*	1.173 ± 0.061
Crude b (1 mg/g)	43.0 ± 2.7	0.953 ± 0.050

Values are presented as mean ± SD.

* P < 0.05.

** P < 0.01.

*** P < 0.001.

a Ultrafiltered hydrolysates;

b Not ultrafiltered hydrolysates.

## Abbreviations

NK: natural killer
ACE: angiotensin-converting enzyme
Con A: Concanavalin A
MTT: 3-(4,5-dimethylththiazoyl-2-yl]-2,5-diphenyltetrazolium bromide
FBS: fetal bovine serum
RP-HPLC: reversed phase high-performance liquid chromatography
CTX: cyclophosphamide
DMSO: dimethyl sulfoxide
